# Medical Education of Females

**Published:** 1853-12

**Authors:** 


### EDITORIAL DEPARTMENT.

---

*Medical Education of Females.*—New projects for good to the race; new ideas of the true mission of its different component parts; and new developments in our ideas of what is right and proper, are constantly thrusting themselves forward for examination.

A few years since, Elizabeth Blackwell presented herself before the faculty of Geneva Medical College, and asked for admission to their lectures. It was granted; the degree of M. D. was conferred upon her, and she crossed the Atlantic for wider opportunities of study.

Thus a new movement was inaugurated. Some enthusiasts took up the cudgels. Woman was to be taken from the quiet of domestic life and thrown into the most laborious of professions, or occupations; the gravest responsibilities which can by any possible conjuncture be imposed upon any individual, were to be entrusted to the feminine intellect, and acts requiring the firmest courage and the strongest physical endurance, were to be her daily task. The gentle amenities of the parlor were to be exchanged for the horrors of the dissecting room; the needle and the scissors for the surgeon's scalpel, the forceps, and the murderous perforator.

With that lack of forethought and judgment, which marks the unthinking reformer, schools for their instruction were instituted on the most superficial and defective plan. The matter-of-fact horrors, the bad odors, the putrefying humanity, the hideous developments of disease, and the necessary disregard of refinement and delicacy, through which the medical student must pass to reach the deeper and more practical truths of medicine, were to be avoided; and anatomy, surgery, obstetrics, and pathology, were to be taught in a lady-like manner.

Soon, the more sensible of those engaged in the movement, recognized the weaknesses of this plan, and foretold the failure which would attend its workings. The plain truth was then placed before the aspirants for female honors. They were told that there was no primrose path to medical science; that female physicians were not desirable unless they were fully prepared to meet the responsibilities of the profession; and we now understand that one of the Female Medical Colleges of Philadelphia, is organized upon a competent and thorough plan of instruction.

This movement involves some considerations of deep interest, not only to the profession, but to the great world of humanity; and at the risk of speaking some disagreeable truths, we shall inquire into their meaning and bearing. The idea of inequality in the sexes, and of positive superiority in the male, is written in every part of God's works, and words. This condition is distinctly and frequently expressed in Holy Writ; and is as plainly enunciated in the physical conformation, and the recorded history of the two sexes.

The prevalence of this idea, or rather the universal recognition of this fact, has, in all nations and ages, given to the two sexes different functions, and positions in society. An inevitable fact always has its reason or final cause. Were woman in any sense the equal of the male, she would have in some age or country acquired an equal supremacy with man. The empire of rule would have been more equally divided. The law holds good everywhere. The distinctions of society are based upon the relative superiority of different classes; and however undemocratic the assertion may be, it is "a self-evident truth, that all men are '*not*' created free and equal." The only equality which can exist, is the mere privilege of acquiring an equality with those who are our superiors. Such an acquisition implies that the person rising, places himself in a condition of superiority to some one else. Even in Heaven this distincton holds good; for we are told that in that happy place itself, "one star differeth from another star in glory." Man is made "a little less than the angels;" archangels are princes in the Heavenly Host, while over all, and above all, is the Godhead.

We have gone farther with the parallel than is necessary. The proposition of woman's inferiority needs no such proof. Her smaller muscular development, her more rotund figure, her feebler will, and keener perception but smaller reasoning capacity, her inability to attend to the active duties of life during the periods of gestation and parturition, all mark out for her a position, not minor in true importance, but inferior in function, and, as we believe, not slavish or unhappy, because just.

These are opinions which, until recently, have been undisputed. But the contingencies incident to a higher civilization, have rendered it necessary that woman should seek some industrial occupations hitherto belonging to the other sex. This necessity is indisputable, and woman is now courageously asserting her right to occupations, to which she is fully competent in her present state of advancement. To the attainment of this end we bid her God-speed.

There is, then, in this movement, the germ of a true and noble reform. The proposition to confer at once upon woman the highest functions of the male sex, is but an excess of a laudable spirit—the result of that fanatic enthusiasm, which, by a conservative law of human nature, is always enlisted in really excellent projects of amelioration. Thus it happens that this unthinking and boyish enthusiasm has proposed to confer upon woman the functions of the physician. Her capacities as a nurse seem to have suggested this project, but he who will give a moment's thought as to what constitutes a good physician, will see that woman does not and cannot possess the qualities necessary.

The cool head, the steady nerve, the sinewy muscle, the facile hand, the power of endurance under great fatigue and loss of sleep without becoming nervous, are the *physical* peculiarities in which woman is lacking. Calmness in danger, with readiness to act boldly and quickly in emergencies, the power to reason rapidly and correctly under distracting influences, the will and mental courage to assume responsibilities unpleasant and injurious to the reputation, are *mental* qualities which she also needs; while the faculty of perception which she possesses in great perfection, will but place before her in the most vivid light the dangers that beset her patient, without the power to analyze, and reason order out of the chaos of perceptions, which crowd and confuse her brain. Hence comes the old saying, that a coward is only a person who sees and appreciates most forcibly the difficulties of his position. A slow but logical thinker is always calmest and safest in danger.

To be placed in a position to which we are incompetent, is the summit of human misery. And in no other occupation can the consciousness of incompetence bring such remorse to the actor, and such deplorable results to the acted upon, as in the medical profession. From such inflictions of that inward sting of conscience, would we wish to protect the sensitive woman. Those are false friends, and foolish advisers, who advise her otherwise. Like a feeble minded but well meaning magistrate, the honors of her position would but illy repay for the anxieties and perplexities of her functions.

But if this movement is to be progressive, if it is not to be checked by the counsels of those best able to advise, then let the thing correct itself. We honor the spirit which actuates Mrs. Sarah J. Hale in this matter. Feeling deeply the embarrassments of the sex in its present organization—probably estimating the female character too highly by knowing her own capacities—she wishes to direct the reform to a practical and useful purpose. Her good sense is too strong to allow her to countenance any half-way education, and looking especially to the vast good which a knowledge of medical science would enable female missionaries of the Christian religion to effect, she is the earnest advocate of a thorough female education.

She would not shun any responsibilities. The dissecting room, the death bed, the loathsome disease, she would have her *eleves* meet firmly and conscientiously, with a view to increased power of doing good. It is unpleasant to prophesy failure to such Christian hopes, such noble purposes; and it is with a feeling of regret that we have penned this article. That no selfish consideration, no jealousy of intrusion on our vested rights, has actuated us, we trust to make evident in what we have yet to say.

The reasons we have assigned as opposed to the propriety of woman's attempting the difficult duties of the physician, are also the reasons why she will not succeed in the attempt. Even at the onset it is conceded that females cannot undertake a country practice. The country doctor must saddle his own horse, and breast such storm and darkness as no woman can endure. These are physical impossibilities which will prevent their accomplishing anything in this field. In the cities they will be placed in competition with the higher minds of the profession, and will necessarily fill a secondary position. It is, therefore, proposed to confine the duties of the female physician to practice in obstetrics, and diseases of women and children; and the large towns are the only fields which they are expected to fill. The practice of obstetrical art, and the treatment of children, require as high a state of medical knowledge as any other branches of the science. Neither are they less fatiguing to the practitioner, nor do their duties require a less amount of learning, tact, and fortitude, than do the other portions of practice.

Another circumstance will militate against their success. Men will not willingly give up their claim of superiority, and the same feeling which prompts the husband and father to employ one of his own sex for his own illnesses, will lead to the employment of the same person in the diseases of his wife and children. To have a different physician for different members of the family, will create estrangement and unpleasant rencounters.

Again, the most specious argument employed by the advocates of female physicians, is the indelicacy of employing a man, in those diseases and conditions incident to her sex. We have already shown that only a man is fully competent, (education being equal,) to the management of these. And the very suggestion of indelicacy, implies a dereliction of duty and gentlemanly feeling on the part of the profession, the existence of which is not proved, and which we indignantly disclaim. Since the inception of male obstetricy, the conduct of physicians has been characterized by such modesty and delicacy of feeling, as does them the highest credit. Neither does the female patient sacrifice any portion of this feeling; and we cannot better express our meaning, than by quoting here the words of a distinguished jurist, in a case where these same questions were involved, and in which the character of the profession was triumphantly vindicated so far as the testimony of witnesses, and the opinions of the bench were concerned. Alluding to this point, Judge Mullett made use of the following beautiful and classic illustration:

"In this submission the fair patient does not discard her delicacy, sensibility, and modesty; these guardians of female virtue may be compelled to step back for the occasion, but they stand around her like Diana's nymphs while she is bathing; and let the practitioner make one significant manifestation of an unholy thought, and they rally around the insulted one, and the wretch is expelled from the confidence he has abused, and ultimately from the profession he has disgraced."

Finally, this matter must be subjected to the test of time and experience. Should it at last be proven that the female does possess the necessary qualifications for a good physician, should she thoroughly and carefully prepare herself for new duties, and faithfully and capably perform them, she will not lack for occupation. It is useless to argue against what are called reform movements. We may, as we in this essay have done, predict as to their success, and philosophize as to their causes of failure, but only the test of time, the necessities of humanity, and the ability on the part of females to meet those necessities, can ever defeat or sustain this project. S. B. H.

